# Emerging therapies for mitochondrial disorders

**DOI:** 10.1093/brain/aww081

**Published:** 2016-05-03

**Authors:** Helen Nightingale, Gerald Pfeffer, David Bargiela, Rita Horvath, Patrick F. Chinnery

**Affiliations:** ^1^ Wellcome Trust Centre for Mitochondrial Research, Institute of Genetic Medicine, Newcastle University, Central Parkway, Newcastle upon Tyne, NE1 3BZ, UK; ^2^ Department of Clinical Neurosciences, University of Calgary, Calgary, Canada; ^3^ Hotchkiss Brain Institute, at the University of Calgary, Calgary, Canada; ^4^ MRC-Mitochondrial Biology Unit, Cambridge Biomedical Campus, Cambridge, CB2 0XY, UK; ^5^ Department of Clinical Neurosciences, Cambridge Biomedical Campus, University of Cambridge, Cambridge, CB2 0QQ, UK

**Keywords:** mitochondrial disorders, treatment, gene therapies, protein, pharmaceuticals

## Abstract

Mitochondrial disorders are a diverse group of debilitating conditions resulting from nuclear and mitochondrial DNA mutations that affect multiple organs, often including the central and peripheral nervous system. Despite major advances in our understanding of the molecular mechanisms, effective treatments have not been forthcoming. For over five decades patients have been treated with different vitamins, co-factors and nutritional supplements, but with no proven benefit. There is therefore a clear need for a new approach. Several new strategies have been proposed acting at the molecular or cellular level. Whilst many show promise *in vitro*, the clinical potential of some is questionable. Here we critically appraise the most promising preclinical developments, placing the greatest emphasis on diseases caused by mitochondrial DNA mutations. With new animal and cellular models, longitudinal deep phenotyping in large patient cohorts, and growing interest from the pharmaceutical industry, the field is poised to make a breakthrough.

## Teaser

Traditionally, mitochondrial disorders have been treated with vitamins, co-factors and nutritional supplements with no proven benefit. While effective treatments are still lacking, several new molecular and cellular strategies have recently been proposed. Nightingale *et al.* critically appraise the most promising preclinical developments.

## Graphical abstract

**Figure:**
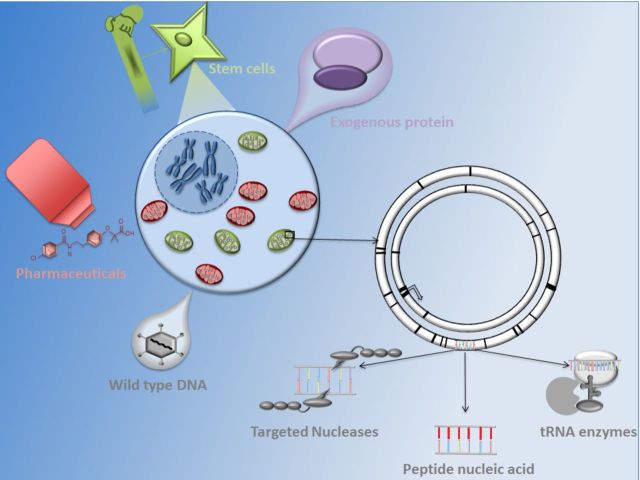
Traditionally, mitochondrial disorders have been treated with vitamins, co-factors and nutritional supplements with no proven benefit. While effective treatments are still lacking, several new molecular and cellular strategies have recently been proposed. Nightingale *et al.* critically appraise the most promising preclinical developments.

## Introduction

Mitochondria are complex intracellular organelles that play a central role in cell homeostasis (Wallace, 1999). They are the principal source of intracellular energy, are intimately involved in both calcium and free radical metabolism, and they can trigger programmed cell death (apoptosis). Tissues and organs that critically dependent on these functions bear the brunt of the pathology in human mitochondrial diseases, which often affect the nervous system, muscle and endocrine organs (Pfeffer *et al.*, 2012). Most mitochondrial disorders are progressive and often result in disability and premature death. Therefore, although they are rare diseases, with a minimum reported prevalence of 1 in 4300 (Schaefer *et al.*, 2004), they have substantial impact on families and healthcare services.

Most mitochondrial disorders are ultimately thought to arise through a bioenergetic defect linked to a deficiency of ATP synthesis. ATP synthesis is the final step of respiration, which is carried out by five oxidative phosphorylation (OXPHOS) complexes situated on the inner mitochondrial membrane. Each complex has multiple protein subunits encoded by two distinct genomes: nuclear chromosomal DNA (nDNA), and the 16.5 kb mitochondrial genome (mtDNA).

Pathogenic mtDNA mutations often cause a subset of classical mitochondrial clinical syndromes (Chinnery and Hudson, 2013). However, an increased number of multisystem mitochondrial disorders are being described in the literature, many of which are yet to be fully characterized clinically and genetically. These include an emerging myriad of nuclear encoded mitochondrial disorders caused by mutations in some of the ∼1500 nuclear genes thought to code for mitochondrial proteins (Chinnery and Hudson, 2013). In the past, the phenotypic and genetic diversity has made clinical diagnosis very challenging. However, with international diagnostic standards (Wolf and Smeitink, 2002), and the widespread availability of molecular genetic techniques, an accurate diagnosis is less challenging than before. Next generation sequencing is revolutionizing the diagnostic approach, with multi-gene panels, whole exome, and whole genome sequencing increasing the pace of diagnosis, and probably reducing the overall costs. As a consequence, more and more patients are being diagnosed with a mitochondrial disorder, placing even greater emphasis on developing treatments.

A recent systematic review identified over 1300 reports using a variety of approaches expected to bypass or enhance components of mitochondrial function. However, the vast majority of these reports are open-labelled case series with less than five subjects. Although ∼30 randomized trials have been carried out to date, no treatment has shown a clear cut benefit on a clinically meaningful end-point (for reviews see Pfeffer *et al.*, 2012; Kerr, 2013). It is therefore likely that components of the traditional ‘mitochondrial cocktail’ do not have a major therapeutic impact on most mitochondrial diseases. There is therefore a clear need for the field to ‘think outside the box’ when developing new treatments, harnessing the massive increase in our understanding of mitochondrial disease pathogenesis. After preclinical evaluation in cellular and animal models, new treatments showing promise should be studied in patients using a rigorous approach (Pfeffer *et al.*, 2013). This review focuses on these new developments, with a particular emphasis on mtDNA diseases, which were previously thought to be intractable. Here we critically appraise each approach, and highlight areas where there is likely to be traction in the future. This is timely, because both small and large pharmaceutical companies are starting to see the potential market in developing treatments for these so-far untreatable disorders.

## Capitalizing on the unique properties of mitochondria

**Figure 1:**
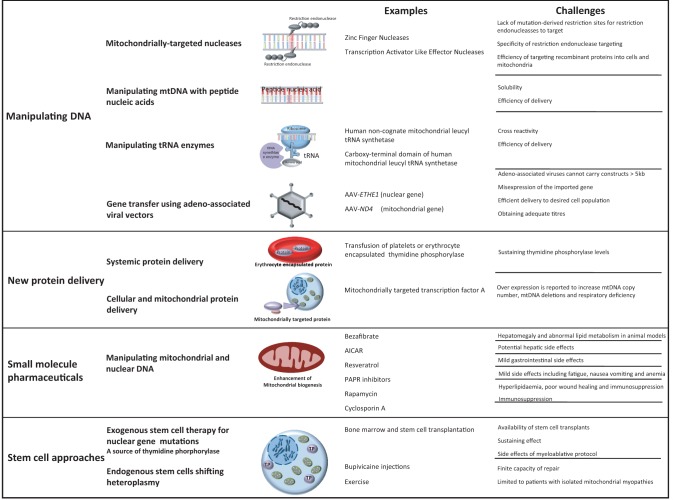
**Overview of novel therapeutic approaches for the treatment of mitochondrial disorders.** AICAR = 5-aminoimidazole-4-carboxamide ribonucleotide; PAPR = poly adenosine diphosphate-ribose polymerase receptor; TP = thymidine phosphorylase.

Mitochondria have unusual characteristics that are potentially targetable for treatment at the molecular level. Mitochondria contain numerous copies of their genome, and most patients with mtDNA disease harbour both mutated and wild-type mtDNA (heteroplasmy) (Wallace, 1999). The proportion of mutated mtDNA needs to be in excess to cause a biochemical defect of the respiratory chain. This critical threshold varies from mutation to mutation, and also between tissues and organs. This partly explains the tissue selectivity and clinical heterogeneity of mitochondrial disorders (Macmillan *et al.*, 1993). The overall amount of mtDNA within a cell is also tightly regulated in a tissue-specific manner, and some tissues (such as skeletal muscle) display a close correlation between mtDNA content and mitochondrial activity (Wang *et al.*, 1999). This complexity raises the possibility of enhancing mitochondrial function by manipulating the ratio of mutant to wild-type mtDNA, or by increasing the amount of wild-type mtDNA. Targeting mutated mtDNA is an alternative approach, although this would need to be highly specific to avoid adverse effects on wild-type genomes. The delivery of wild-type nDNA (Di Meo *et al.*, 2012; Bottani *et al.*, 2014; Torres-Torronteras *et al.*, 2014) or mtDNA (Ellouze *et al.*, 2008; Yu et al., 2012a,b) using viral vectors is another possibility, or perhaps the replacement of dysfunctional proteins via the cell nucleus, hitch-hiking on the mitochondrial import mechanism (Perales-Clemente *et al.*, 2011). Moving away from the two genomes, small molecule screens may enhance function of the respiratory chain, stem cell therapies could correct enzyme defects due to nuclear gene defects (Hirano *et al.*, 2006; Hill *et al.*, 2009; Halter *et al.*, 2011; Lenoci *et al.*, 2011; Filosto *et al.*, 2012; Sicurelli *et al.*, 2012; Hussein, 2013), and treatments aimed at non-specifically preventing neurodegeneration may be the way forward. Some of these approaches capitalize on the ‘uniqueness’ of mitochondria, where other approaches build on knowledge acquired from rare and common neurological disorders. Having an open mind is critical at this stage—the first effective treatment may not come from an obvious place. This review considers each, structured into four sections (Fig. 1): manipulating DNA; new protein delivery; small molecule pharmaceuticals; and finally, stem cell approaches. Our discussion focuses on the likelihood of these treatments being used in the clinic for patients with mitochondrial disease, and does not discuss recent work aimed at preventing these disorders.

## Nucleic acid-based approaches

Several approaches, described below, have been developed to manipulate or replace mutated nDNA and mtDNA. As for all potential therapies, the multi-organ nature of mitochondrial disorders and difficulties of transferring therapies across cellular and mitochondrial membranes without causing toxic effects (Mingozzi and High, 2011), makes therapeutic targeting difficult. However, as explored further below, methods exist to overcome these difficulties including the use of viral vectors (Mingozzi and High, 2011), harnessing allotopic expression (nuclear expression of mtDNA encoded protein) (Farrar *et al.*, 2013), or fusion of therapeutic molecules to targeting proteins (Iyer *et al.*, 2009).

### Mitochondrially-targeted nucleases

Restriction endonuclease approaches were developed to recognize specific DNA sequences, produce double-stranded DNA breaks, and thereby initiate target molecule degradation. Unlike other gene therapy approaches, restriction endonucleases are not contingent on stable vector integration into the nuclear genome. In theory, a single treatment could induce a stable modulation of mtDNA heteroplasmy, correcting the biochemical defect within diseased cells (Minczuk *et al.*, 2008; Bacman *et al.*, 2013; Gammage *et al.*, 2014). Restriction endonucleases recognize a broad repertoire of mtDNA sequences (‘restriction sites’) and some pathogenic mtDNA mutations add restriction sites that restriction endonucleases specifically target.

The m.8993T > G mtDNA mutation causes Leigh syndrome or neuropathy ataxia retinitis pigmentosa, and mitochondrially-targeted restriction endonuclease SmaI has been shown to selectively eliminate m.8993T > G from patient-derived cybrid cells. Over time, the treatment resulted in repopulation with wild-type mtDNA, and normalization of cellular ATP content (Tanaka *et al.*, 2002).

**Figure 2:**
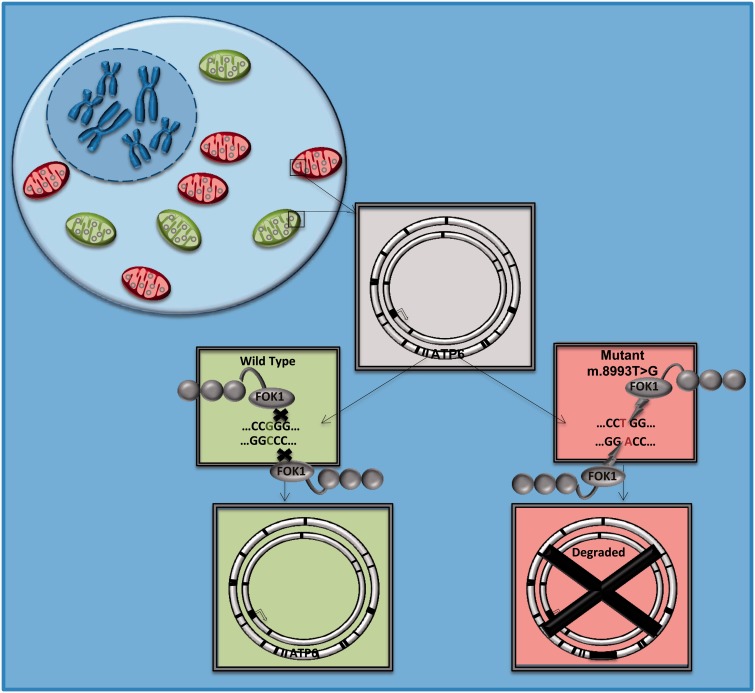
**Endonucleases.** Endonucleases are used to target specific sequences in mtDNA causing double-strand breaks and degradation of mtDNA. For example the endonuclease ZFN has been shown to reduce mutation load in a cybrid model of Leigh and NARP syndrome, which are caused by the mtDNA mutation m.8933T > G within the ATP6 domain. ZFN binds specifically to the mutant form of the mtDNA and the FOK1 endonuclease domain cleaves the DNA molecule, which is then degraded.

Unfortunately, a key limitation of restriction endonucleases is that very few human pathogenic mutations create restriction sites amenable to this form of targeted destruction. This can in part be overcome by restriction endonucleases custom designed to bind specific DNA sequences (Klug, 2010). A zinc finger nuclease (ZFN) consists of tandem repeat zinc fingers, each binding approximately three DNA bases, combined with a FokI endonuclease domain functioning as the DNA cleavage module (Klug, 2010). Specificity is achieved through different combinations of zinc fingers. ZFNs have also been shown to selectively eliminate the m.8993T > G mutation in a cybrid model (Fig. 2) (Minczuk *et al.*, 2008). Early ZFNs caused significant cytotoxicity, homodimerization (Ramalingam *et al.*, 2011) and off-target binding. This would be detrimental *in vivo*, thereby limiting their clinical use (Radecke *et al.*, 2010), but are being addressed by improved design (Gammage *et al.*, 2014). The architecture of dimeric ZFNs ensures that the active cleavage reagent is only assembled at desired restriction sites, overcoming targeting difficulties and concerns about constitutive nuclease activity (Gammage *et al.*, 2014). Customized ZFNs targeted to mitochondria cause shifts in heteroplasmy through the selective degradation of mtDNA containing the m.8993T > G point mutation, and the large scale (4977 bp) mtDNA ‘common deletion’, which is the most common cause of chronic progressive external ophthalmoplegia, Kearns-Sayre syndrome, and Pearson marrow pancreas syndrome (Gammage *et al.*, 2014). This work opens up the opportunity to develop a library of bespoke ZFNs against more common pathogenic mtDNA mutations, but the shifts in heteroplasmy have been limited to date. Longer-term studies, particularly using animal models, will hopefully show that ZFNs can improve biochemical function *in vivo—*but this will be technically demanding, not least because of the challenges delivering these agents at an appropriate concentration to affected tissues.

**Figure 3:**
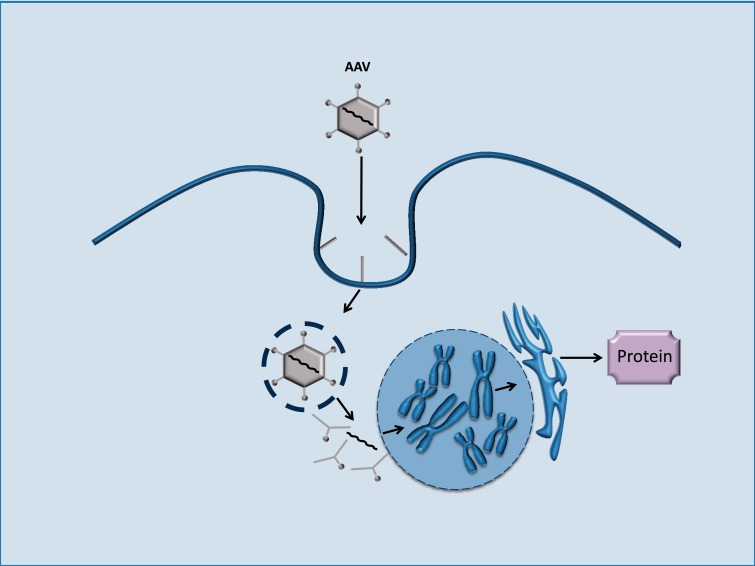
**Adeno-associated viral vectors expressing wild-type gene constructs.** Gene constructs can be introduced into host cells by AAV and transcribed within the nucleus of the host. The end product is a functional protein, which can replace or bypass dysfunctional proteins resulting from mutations in the host’s nDNA or mtDNA.

When combined with a non-specific nuclease (e.g. FokI), TALEs (transcription activator-like effectors) become target-specific DNA nucleases (TALENs) (Christian *et al.*, 2010). TALENs are potentially much more potent than ZFNs, but their larger size limits their use with adeno-associated viral vectors (AAVs) (Ellis *et al.*, 2013), TALEs contain repetitive domain modules, each of which bind individual nucleotides (Moscou and Bogdanove, 2009), thus different domain module combinations can be used to ensure specificity.

A recent report described the use of mitochondrially localized TALENs targeted for mtDNA species with the ‘common deletion’ (m.8483_13459del4977), and a separate TALEN that recognizes a point mutation (m.14459G > A in the *MT-ND6* gene) in an osteosarcoma cybrid cell model (Bacman *et al.*, 2013). The TALEN markedly reduced the level of deleted mtDNA species allowing compensatory mitochondrial biogenesis to preferentially restore wild-type mtDNA (Bacman *et al.*, 2013). In studies with the point mutation, relative specificity was demonstrated for the mutant over the wild-type sequence (Bacman *et al.*, 2013). Interestingly the heteroplasmy shift persisted after the TALENs were no longer detectable, suggesting long-lasting effects after a single treatment (Bacman *et al.*, 2013). However, the impact of the rapid reduction in mtDNA copy number following TALEN treatment raises serious concerns about their potential toxicity, not least because several fatal mitochondrial diseases are caused by mtDNA depletion. However, the co-induction of mitochondrial biogenesis could circumvent this issue, but will need careful evaluation in animal models before any clinical studies.

The most recent addition to this group of endonucleases is Cas9 nuclease. Unlike the ZFN and TALE proteins that target the FokI endonuclease, Cas9 is targeted using a much smaller short RNA sequence CRISPR (Hsu *et al.*, 2014). This RNA sequence can more easily be altered to change target site than for TALEs and distinct RNA sequences can be used target Cas9 to induce multiple double-stranded DNA breaks (Hsu *et al.*, 2014; Cox *et al.*, 2015). Furthermore this system is not hindered by context-specific binding reported for ZFNs and TALENs (Hsu *et al.*, 2014; Cox *et al.*, 2015). Although there has been controversy surrounding this approach, recently Cas9/CRISPR has been demonstrated to restore the weight loss phenotype of a mouse model of fatal disease hereditary tyrosinaemia type 1 (Yin *et al.*, 2014).

The potential use of restriction endonucleases to prevent germline transmission has also been investigated. MitoTALENs have been demonstrated to reduce levels of human mutated mtDNA responsible for Leber hereditary optic neuropathy (LHON) and NARP (neurogenic muscle weakness, ataxia, and retinitis pigmentosa) in oocytes (Reddy *et al.*, 2015).

### Peptide nucleic acids

Sequence-specific peptide nucleic acids selectively bind mutant mtDNA and induce direct mtDNA strand degradation (Mukherjee *et al.*, 2008). Although conjugation with mitochondrial targeting peptides promotes importation and successful targeting into human cells in culture (Chinnery *et al.*, 1999), this initially failed to modulate heteroplasmy in patient-derived cell lines (Kyriakouli *et al.*, 2008). In part this is due to a failure to transmit the peptide nucleic acids across the mitochondrial membrane, so techniques of traversing the cell membrane are being explored, such as ‘cell membrane crossing oligomers’ (Kyriakouli *et al.*, 2008), which possess greater polarity, and RNA vectors (Comte *et al.*, 2013). Although these examples show promise, evidence for therapeutic benefit remains sparse despite nearly two decades of research. Targeted delivery will be a common problem, and potential toxic effects need to be excluded in long-term animal studies. Again, limited availability of animal models of human mtDNA diseases has hindered progress.

### Manipulating tRNAs

The large array of pathogenic mtDNA mutations affecting tRNA genes prompted the exploration of tRNA-targeted therapies (Yarham *et al.*, 2011). Of particular interest are tRNA synthetases that catalyse the addition of specific amino acid molecules to cognate tRNA molecules during protein translation. Early work demonstrated that the overexpression of cognate aminoacyl mt-tRNA synthetase stabilized mt-tRNA (mitochondrial tRNA) molecules (Rorbach *et al.*, 2008). More recently overexpression of human non-cognate mitochondrial leucyl tRNA synthetase and its small carboxy-terminal domain were shown to partially rescue the biochemical dysfunction secondary to mt-tRNA defects (Hornig-Do *et al.*, 2014; Perli *et al.*, 2014).

### Gene transfer using adeno-associated viral vectors

Harnessing viral vectors to transfer wild-type genes into the cell nucleus dates back to the late 1990’s. Although initial excitement was tempered by the death of a patient in an early clinical trial (Raper *et al.*, 2003), much progress has been made in developing new, less immunogenic vectors such as AAV (Wang *et al.*, 2012) (Fig. 3).

### Gene therapy for nuclear-mitochondrial disorders

Ethylmalonic encephalopathy classically has heterogeneous phenotypes with neurological, gastrointestinal, metabolic and psychiatric sequelae (Tiranti *et al.*, 2004) and results from mutations in the *ETHE1* gene (Tiranti *et al.*, 2004). *ETHE1* encodes a ubiquitous mitochondrial sulphur dioxygenase that detoxifies hydrogen sulphide (Tiranti *et al.*, 2004). AAV-mediated hepatic *ETHE1* gene expression restores sulphur dioxygenase activity in a murine model of ethylmalonic encephalopathy, correcting biochemical abnormalities within liver, muscle, and brain; and increasing survival from a few weeks to more than 6 months (Di Meo *et al.*, 2012). However, targeting human skeletal muscle and other large tissues is extremely challenging, given currently achievable viral titres. Targeting the relatively impenetrable blood–brain barrier presents another hurdle in the treatment of nuclear-encoded mitochondrial diseases. However, there are exceptions where the approach has real potential for therapeutic benefit in the near future.

Mitochondrial neurogastrointestinal encephalopathy (MNGIE) is a rare defect of the thymidine phosphorylase encoding gene *TYMP* (Nishigaki *et al.*, 2003). Mutations in *TYMP* cause high circulating levels of thymidine, which in turn lead to mtDNA depletion and the formation of secondary mtDNA deletions and point mutations. Treating a single organ, such as the liver, has been proposed to generate enough enzyme to ‘detoxify’ the systemic circulation and thereby prevent disease progression (Torres-Torronteras *et al.*, 2014). In keeping with this idea, the permanent and dose-dependent reduction of systemic toxic metabolite levels in a murine model of MNGIE has been demonstrated with the construct, AAV2/8-hc*TYMP*, which possesses a liver-specific promoter (Torres-Torronteras *et al.*, 2014). Interestingly, recent evidence that the liver can synthesize potentially therapeutic thymidine phosphorylase levels has prompted the suggestion that liver transplantation would be an effective therapy for MNGIE (Boschetti *et al.*, 2014).

The Harlequin mouse model of mitochondrial complex 1 deficiency results from a pro-viral X-linked gene insertion causing severely reduced apoptosis-inducing factor 1 (AIF1) expression. These mice exhibit retinal and optic nerve respiratory chain complex 1 deficiency causing glial and microglial cell activation, retinal ganglion loss and optic atrophy. The intra-vitreal administration of an AAV2/2_AIF1 vector has been shown to counteract these changes (Bouaita *et al.*, 2012).

Finally, mutations in the *MPV17* gene result in a hepatocerebral mtDNA depletion syndrome that is also potentially amenable to gene therapy (Bottani *et al.*, 2014). *MPV17* knockout (KO) mice exhibit hepatic mtDNA depletion and liver failure when subjected to a ketogenic diet (Bottani *et al.*, 2014). This phenotype is rescued by AAV viral vector expression of human *MPV17* cDNA with a hepatic specific promoter (Bottani *et al.*, 2014). However, it is unclear whether the tissue-specific expression of the transgene will correct the cerebral disorder. This is critical because the CNS features of the disorder are fatal.

### Gene therapy for mitochondrial DNA disorders

Delivering gene therapy into mitochondria presents an even greater challenge. Many cells vulnerable to mitochondrial disease contain thousands of mitochondria, and the mitochondrial membrane is relatively impermeable. There is little evidence that AAVs penetrate into the mitochondrial matrix, so achieving the high titres required to intercalate with multiple copies of mtDNA seems almost insurmountable at present. An alternative approach is to harness well-established techniques for nuclear gene therapy to ‘allotopically express’ mtDNA encoded proteins. If carefully engineered, and with a targeting peptide presequence, this has the potential to deliver wild-type proteins to the mitochondrial membrane.

After early *in vitro* work expressing the subunits of the mitochondrial ATPase (Bokori-Brown and Holt, 2006), several laboratories have focused on the allotropic expression of complex I subunits with a view to treating the most common mtDNA disorder: LHON. The mtDNA m.11778G > A mutation affects nicotinamide adenine dinucleotide (NADH) dehydrogenase subunit 4 (ND4 of complex 1, and accounts for ∼50% of LHON patients). Not surprisingly, this mutation has been the focus for most of the studies to date. *In vitro* and preclinical work (Guy *et al.*, 2002; Qi *et al.*, 2007; Ellouze *et al.*, 2008; Yu *et al.*, 2012*a*,*b*) provide some evidence base for this approach, and preparations for early phase clinical (Lam *et al.*, 2010; Bin, 2015; Vignal, 2015) studies are well under way in the USA, China and Europe.

The allotopic expression of a synthetic *ND4* gene is reported to increase ATP synthesis in a human cell harbouring the m.11778G > A mutation (Guy *et al.*, 2002). More recently, in murine LHON models (Qi *et al.*, 2007; Ellouze *et al.*, 2008; Yu *et al.*, 2012*a*,*b*), intraocular injection of human nuclear *ND4* gene constructs expressed by AAV has been shown to be safe (Ellouze *et al.*, 2008; Yu *et al.*, 2012*a*); non-mutagenic to host mtDNA (Yu *et al.*, 2013); prevent retinal ganglion cells loss; and improve optic atrophy and vision (Ellouze *et al.*, 2008; Yu *et al.*, 2012*a*). However, as explored further below, this contradicts the longstanding evidence of interspecies protein incompatibility (McKenzie *et al.*, 2003; Perales-Clemente *et al.*, 2011). Furthermore, although the allotopically-expressed proteins appear to be imported, it is not clear whether the new peptides integrate within the respiratory chain (Figueroa-Martinez *et al.*, 2011). Indeed, one study demonstrated that mitochondrially-encoded NADH (reduced nicotinamide adenine dinucleotide) dehydrogenase (with an additional mitochondrial transduction domain) was expressed in the nucleus and partially rescued the phenotype of cells with a homoplasmic knockout mutation for *mt-Nd6* (Perales-Clemente *et al.*, 2011). However, the expressed protein seemed to lie largely outside the outer mitochondrial membrane (Perales-Clemente *et al.*, 2011). Despite these concerns, groups in Miami (Lam *et al.*, 2010) and China (Bin, 2015) are currently recruiting for a trial to examine intraocular AAV-*ND4* for LHON. A further safety trial is currently recruiting in France (Vignal, 2015).

### Expression of non-mammalian genes

There is interest in using AAV expressing mature yeast complexes (not troubled by issues of integration) to bypass mutations in multi-subunit mammalian equivalents. This ‘transkingdom approach’ has been shown to preserve retinal ganglion cells, improve optic nerve function and improve vision in a rotenone-induced LHON rodent model (Marella *et al.*, 2010; Chadderton *et al.*, 2013). Lentiviral vectors expressing a yeast alternative oxidase have also been demonstrated to rescue COX (cytochrome *c* oxidase) deficiency in a mouse model (El-Khoury *et al.*, 2013).

Despite the emerging evidence supporting transkingdom protein expression, it remains a contentious issue. The co-evolution of nDNA and mtDNA has led to species-specific genome compatibility (Kenyon and Moraes, 1997). Incompatibilities between nDNA and mtDNA explain why Human-Gorilla xenomitochondrial cybrids have a respiratory chain deficiency (Barrientos *et al.*, 1998), and incompatibles of interspecies mtDNA gene products (McKenzie *et al.*, 2003; Perales-Clemente *et al.*, 2011), suggest that caution is needed with this approach, which is very much at the early preclinical phase.

### Improving mitochondrial DNA copy number in mitochondrial DNA depletion syndromes

*In vitro* and preclinical evidence support the therapeutic potential of increasing deoxyribonucleotides to treat rate autosomal recessive mtDNA depletion syndromes. One approach involves deoxyribonucleoside supplementation, which increased mtDNA levels (copy number) in primary cell cultures from patients with deoxyguanosine kinase deficiency due to *DGUOK* mutations (Bulst *et al.*, 2009; Camara *et al.*, 2014). A similar effect has also been seen in an *in vitro* model of MNGIE (Camara *et al.*, 2014). Another strategy involves inhibition of deoxyribonucleoside catabolism, which has also been shown to improve mtDNA copy number *in vitro* (Camara *et al.*, 2014). More recently, deoxyribonucleoside supplementation in a knock-in mouse model of thymidine kinase 2 (*TK2*) deficiency increased mtDNA copy number, improved mitochondrial respiratory chain function, and prolonged life span (Garone *et al.*, 2014).

## New protein delivery

### Systemic protein delivery

The identification of a specific protein dysfunction or deficiency resulting in mitochondrial disease has led to the possibility of protein replacement or the removal of accumulated toxic metabolites.

In MNGIE, haemodialysis has been used to remove toxins (Spinazzola *et al.*, 2002) and the transfusion of platelets (Lara *et al.*, 2006) or erythrocyte-encapsulated thymidine phosphorylase (EE-TP) has been explored as a means of delivering exogenous thymidine phosphorylase (Table 2) (Moran *et al.*, 2008; Bax *et al.*, 2013; Hussein, 2013). At present there is no convincing evidence of sustained clinical benefit from either approach. However, drawing on experience from other inborn errors of metabolism such as adenosine deaminase deficiency (Bax *et al.*, 2007), direct enzyme replacement could proceed into early phase studies in the short to medium term. Indeed, a recent preclinical trial has demonstrated adequate safety of recombinant EE-TP in mice and beagle dogs (Levene *et al.*, 2013). However, the reported antibody generation (Levene *et al.*, 2013) may preclude long-term use, and based on experience with other rare inherited enzyme defects, this approach is likely to be extremely expensive.

**Table 1:** Exogenous stem cell therapy for nuclear gene mutations in MNGIE

**Reference**	**Age/Gender**	**Intervention**	**Outcome measures**	**Outcome**	**Reported adverse events**
Hussein 2013	Patient 1: M/24	HLA fully matched BMT	TP activity	TP activity initially rose from 1 to 180 nmol/h/mg/protein but then dropped to 10 nmol/h/mg/protein 10 months post-transplantation.	GVHD
			dThd and dUrd levels	Normalized.	
			Clinical condition	Subjective improvement in walking, hearing, abdominal pain, dysphagia, vomiting and diarrhoea and weight gain 3 months post-transplantation. No improvement in peripheral neuritis or foot drop.	
Filosto *et al.*, 2012	Patient 1: F/34	HLA matched HSCT	TP activity	Patient 1 and 2: TP activity rose from 0 nmol/h/mg/protein to within normal range.	Patient 1: Pyrexia of unknown origin, hyperglycaemia, pancreatitis, CMV reactivation, GVHD and complications of immunodeficiency.
	Patient 2: F/22		Clinical condition	Patient 1: no improvement in neurological assessment, nerve conduction studies or gastrointestinal symptoms. Patient died 15 months post-transplantation.	Patient 2: mild GVHD. Posterior reversible encephalopathy syndrome and *C. difficile* diarrhoea, followed by septic shock and ARDS.
				Patient 2: reduced gastrointestinal discomfort but no improvement in neurological symptoms. Died 8 months post-transplantation.	
Sicurelli *et al.*, 2012	Patient 1: F/23	HLA matched HSCT	TP activity	TP activity rose from 0 nmol/h/mg/protein to within normal range.	Worsening of sensory neuropathy.
			dThd and dUrd levels	dThd and dUrd levels normalized.	
			Blood lactate	Blood lactate decreased from 2.3 to 1.5 mmol/l.	
			Clinical condition	Resolution of diarrhoea, vomiting and pain and fatigability 1 month post-transplantation. MRC muscle strength score increased by 16 points and proximal motor conduction velocities improved 12 months post-transplantation. Sensory neuropathy worsened and MRI defined leukoencephaolopathy was unchanged.	
Hirano *et al.*, 2006	Patient 1: F/21	HLA matched placental cord blood transplant	TP activity	Patient 1 and 2: TP activity increased to peaks of 104 and 165 nmol/h/mg/protein after 1-2 months in patient 1 and 2 respectively but then decreased.	Patient 1: non-engraftment.
	Patient 2: F/30	HLA matched related SCT	dThd and dUrd levels	Patient 1 and 2: dThd and dUrd levels were reduced in both patients and to within normal range for patient 2 after 2 months.	
			Clinical condition	Patient 1: No symptomatic improvement was documented. Died 86 days Post-transplantation from disease progression.	
				Patient 2: subjective improvement in abdominal pain, swallowing and distal limb numbness 6.5 months post-transplantation. Biceps and ankle reflexes returned	
Lenoci *et al.*, 2011 (abstract only)	Patient 1: F/21	HLA matched related HSCT	TP activity	TP activity normalized 55 days post-transplantation.	None reported.
			dThd and dUrd levels	dThd and dUrd levels decreased.	
			Clinical condition	Subjective improvement in vomiting, diarrhoea and abdominal pain with increase in body weight. No improvement in neurological symptoms was documented.	
Hill *et al.*, 2009	Patient 1: F/41	HLA matched related peripheral blood SCT	TP activity	TP activity normalized by day 25 post-transplantation and was still normal 14 months post-transplantation.	Idiopathic thrombocytopenic purpura and gastrointestinal haemorrhage.
			Clinical condition	Improvement in oral intake and increase in weight. Neurological performance status, repeat EMG and cerebral MRI scan were unchanged.	

A series of case reports of stem cell therapies in eight patients with MNGIE showed variable results. Overall thymidine phosphorylase activity tended to increase post-transplantation and some improvements in gastrointestinal and neurological symptoms were reported.

ARDS = acute respiratory distress syndrome; BMT = bone marrow transplant; CMV = cytomegalovirus; EMG = electromyogram; F = female; GVHD = graft versus host disease; HLA = human leukocyte antigen; HSCT = haematopoietic stem cell transplantation; M = male; MRC = Medical Research Council; SCT = stem cell transplant; TP = thymidine phosphorylase; dTHd = thymidine; dURD = deoxythymidine.

**Table 2:** New protein delivery to treat MNGIE

**Reference**	**Age/gender**	**Intervention**	**Outcome measures**	**Outcome**	**Reported adverse events**
Bax *et al.*, 2013	M/28	Encapsulated TP	Plasma dThd and dUrd levels between cycles	Thd and dUrd levels reduced to 8.1 and 12.6 mmol/l from 20.5 and 30.6 mmol/l, respectively after 27 cycles.	Mild transient reaction to infusion with coughing and head and neck erythema.
			Urinary dThd and dUrd levels between cycles	Urinary dThd and dUrd levels reduced to 192–282 and 0.0–184 mmol/24 from 421 and 324 mmol/24 h, respectively from cycle 21.	
			Plasma creatine kinase	Creatine kinase level reduced from 1200 U/l pre-therapy to 254 U/l (normal range 40–320 U/l) at 23 months.	
			Clinical condition	Increase in MRC power sum score 56 (baseline) to 74 (23 months post-infusion). Improvements in gait, balance, sensory ataxia and finger dexterity. Weight increased from 57.4 kg (baseline) to 61.2 kg (post-infusion). No change in EMG or nerve conduction studies. Patient self reported an increased walking distance from 1 to 10 km.	
Moran *et al.*, 2008	F/21	Encapsulated TP	Plasma dThd and dUrd levels	3 days post-infusion plasma dThd and dUrd levels reduced (but not to within normal range) but then began to rise.	
			Urinary dThd and dUrd levels	3 days post-infusion urinary dThd and dUrd levels fell to 6% and 13% of the pre-therapy values respectively (these values were still not within normal range).	None reported.
			Clinical condition	Clinical condition remained poor and the patient died 21 days post-infusion.	

Systemic protein delivery: thymidine phosphorylase (TP) encapsulated in erythrocytes. Thymidine phosphorylase can be encapsulated in autologous blood erythrocytes by reversible hypo-osmotic dialysis, these erythrocytes can then used for infusion to increase the functional thymidine phosphorylase levels of the recipient. Results from two case reports of infusion of thymidine phosphorylase encapsulated in erythrocytes for patients with MNGIE demonstrated initial reductions in toxic product levels post transplantation and some reported clinical improvement. MRC = Medical Research Council; dTHd = thymidine; dURD = deoxythymidine.

### Cellular and mitochondrial protein delivery

Delivering proteins across the mitochondrial membrane is challenging because of their hydrophobicity, but harnessing endogenous mitochondrial import machinery provides a solution.

The mitochondrial protein TFAM (transcription factor A, mitochondrial) is important for mtDNA expression and replication (Iyer *et al.*, 2009). Treating LHON cybrids with mitochondrially-targeted TFAM increased respiratory chain protein levels and enhanced cellular respiration (Iyer *et al.*, 2009). Furthermore, the systemic injection of this construct into mice increased aerobic respiration in muscle and brain, and improved overall motor endurance (Iyer *et al.*, 2009). However, caution is needed when considering translation into human trials because transgenic mice overexpressing TFAM show increased mtDNA copy number, mtDNA deletions and respiratory chain deficiency—all of which could lead to long-term toxicity (Ylikallio *et al.*, 2010). This work highlights the difficulty in interpreting the results from different cellular and animal models that can be misleading. Studying a new treatment in a range of cellular and animal systems will reduce the chance of inappropriately rejecting a treatment based on adverse effects in one model. On the other hand, studying more than one model will reduce the chance of pursuing a drug that will never make it into clinical use.

## Small molecule pharmaceuticals

Future pharmaceutical development focusing on disease-specific or patient-specific molecular targets aimed at boosting residual mitochondrial function is likely to be more successful than previous approaches, which were generally based on a non-specific bypass or amelioration of defective components of the respiratory chain.

Systematic screening of small molecules has recently increased in popularity, in part driven by the widespread availability of patient cell lines, and technological advances in medium-to-high throughput screening (Golubitzky *et al.*, 2011; Pfeffer *et al.*, 2013; Soiferman *et al.*, 2014). Although the complexity of the diverse genotype–phenotype relationship still complicates drug discovery (Macmillan *et al.*, 1993; Wallace, 1999; Wang *et al.*, 1999; Koopman *et al.*, 2012), this could be circumvented by screening a panel of drugs on specific patient cell lines, leading to a personalized or precision medicine approach (Koopman *et al.*, 2012), which can be optimized for testing in animals models then clinical studies (Koopman *et al.*, 2012). So called ‘N-of-one’ clinical trials provide one way of moving forward in patients, proving the drugs have a measurable effect in a short time period.

### Induction of mitochondrial biogenesis

#### Bezafibrate

**Figure 4:**
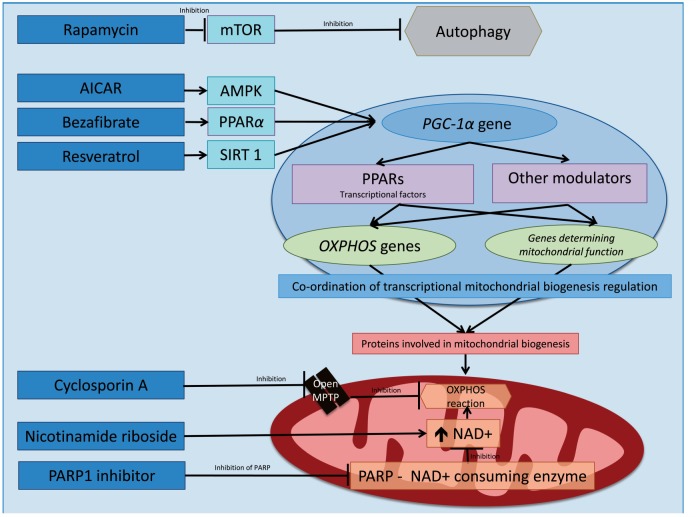
**Schematic representation of pharmaceutical modulators of mitochondrial biogenesis.** There are multiple signalling pathways involved in mitochondrial biogenesis. PGC-1α (encoded by *PPARGC1A*), which is a co-activator for a family of transcriptional factors known as PPARs, co-ordinates via a cascade of nuclear encoded proteins the vast majority transcriptional mitochondrial biogenesis. Novel pharmacological therapies aim to modulate PCG-1α mtDNA expression (e.g. PPARα) and protein expression or target downstream pathways. Bezafibrate is pharmacological ligand for the transcriptional co-factor PGC-1α. AICAR activates AMP-activated protein kinase (AMPK) and is thought to modulate increased mitochondrial biogenesis through PGC-1α. The natural polyphenol resveratrol activates sirtuin 1 (SIRT1). Sirtuins are part of a group of oxidizing NAD-dependent protein deacetylases. Upon activation, for example, by PGC-1α or transcription factor A, mitochondrial (TFAM) they promote mitochondrial respiratory chain activities and the transcription of genes modulating mitochondrial biogenesis and function. Nicotinamide riboside can be used to supplement NAD+ levels. PARP1 functions as a NAD+ consuming enzyme. Thus in turn inhibition of PARP1 has been demonstrated to increase NAD+ bioavailability and SIRT1 activity (not shown above) promoting oxidative phosphorylation. Rapamycin inhibits mTOR, which in turn releases mTOR inhibition of autophagy. Cyclosporin A inhibits the mitochondrial permeability transition pore (MPTP). Opening of the mitochondrial permeability transition pore is thought to deplete pyridine nucleotides thus impairing mitochondrial oxidative respiration.

Several studies have focussed on peroxisome proliferator-activated receptor (PPAR) gamma co-activator 1 alpha (PGC-1α, encoded by *PPARGC1A*) as a potential treatment for mitochondrial disease. PGC-1α co-ordinates the vast majority transcriptional mitochondrial biogenesis regulation in accordance with metabolic demand (Scarpulla *et al.*, 2012). The array of novel pharmaceuticals discussed below either modulate PCG-1α mtDNA or protein expression or target its downstream pathways (Fig. 4). Bezafibrate is pan-agonist for the PPAR family of transcriptional factors. PPAR-α activation promotes mitochondrial biogenesis by upregulating *PPARGC1A* gene expression (Kanabus *et al.*, 2014). Although bezafibrate was reported to increase COX activity and ameliorate the disease phenotype in a muscle-specific *Cox10* knockout mouse (Wenz *et al.*, 2008), this observation has been retracted. Bezafibrate was not effective in two other murine models of COX deficiency *Surf1* knockout (Viscomi *et al.*, 2011) or Deletor mice (Yatsuga and Suomalainen, 2012). Bezafibrate induced hepatomegaly and abnormal lipid metabolism is reported in *Surf1* knockout (Viscomi *et al.*, 2011) and Deletor mice (Yatsuga and Suomalainen, 2012). Importantly this does not appear to occur at comparable doses in humans.

#### AICAR

5-Aminoimidazole-4-carboxamide ribonucleotide (AICAR) is an adenosine monophosphate (AMP) analogue (Merrill *et al.*, 1997) causing AMPK to initiate catabolic mechanisms to produce ATP and inhibit anabolic processes (Canto and Auwerx, 2009), promote *PPARGC1A* gene transcription (Terada *et al.*, 2002) and PGC-1α activity by direct phosphorylation (Jager *et al.*, 2007). AICAR has been shown to increase mitochondrial biogenesis and ATP production alongside decreasing reactive oxygen species human complex I deficient fibroblasts (Golubitzky *et al.*, 2011). AICAR also causes partial correction of COX defects in mice (Viscomi *et al.*, 2011). The low apparent toxicity in humans supports its further investigation (Dixon *et al.*, 1991), although some caution is needed as increased liver weight is suggested with prolonged use in mice (Winder *et al.*, 2000).

#### Resveratrol

Resveratrol is a natural polyphenol, known to increase NAD+ (oxidized nicotinamide adenine dinucleotide) levels, which activate the protein deacetylase SIRT1 (Kanabus *et al.*, 2014). SIRT1 activates PGC-1α by deacetylation sparking a chain of signalling pathways promoting mitochondrial biogenesis and function (Canto and Auwerx, 2009; Kanabus *et al.*, 2014).

Resveratrol activation of SIRT1 has been demonstrated to restore normal function in human fibroblasts with inborn errors of mitochondrial fatty acid β-oxidation (Bastin *et al.*, 2011). Such preclinical data have prompted investigation of resveratrol for diseases with mitochondrial pathogenesis such as Friedreich’s ataxia (Delatycki, 2012; Yiu *et al.*, 2013).

A recently completed open-label clinical pilot study investigating resveratrol in Friedrich’s ataxia has presented preliminary data (full publication is awaited) showing improvement in disease severity and oxidative stress markers (Delatycki, 2012; Yiu *et al.*, 2013). Resveratrol is reported to have only mild gastrointestinal side effects (Patel *et al.*, 2011).

### Modulating NAD+ bioavailability

The balance between NAD+ and NADH is crucial to the process of oxidative phosphorylation. With increased NAD+ bioavailability enhancing oxidative phosphorylation. Indeed NAD+ supplementation with nicotinamide riboside has produced promising biochemical and clinical improvements in two mitochondrial myopathy murine models; a nuclear gene (*Sco2*) knockout/knockin mouse (Cerutti *et al.*, 2014), and the Deletor mouse possessing a nuclear gene mutation resulting in mtDNA deletions (Khan *et al.*, 2014).

### PARP inhibitors

Inhibition of the NAD+-consuming enzyme poly ADP (adenosine diphosphate-ribose) polymerase 1 (PARP1) increases NAD+ bioavailability, SIRT1 activity and subsequently oxidative metabolism (Bai *et al.*, 2011). There is convincing evidence from murine models of myopathy (Cerutti *et al.*, 2014) and mitochondrial encephalopathy (Felici *et al.*, 2014), of the potential benefit of PARP inhibition in human mitochondrial disease. Toxicity is also reported to be low (Tutt *et al.*, 2010).

### Inhibiting cytosolic translation and autophagy

During starvation states damaged cellular components are degraded to maintain cellular activity and viability (Kim *et al.*, 2011). This process termed autophagy is inhibited by mammalian target of rapamycin (mTOR) (Kim *et al.*, 2011).

Administration of the mTOR inhibitor rapamycin in a murine model of Leigh syndrome delayed the onset of symptoms, reduced neuroinflammation and prevented brain lesions, leading to an increased life span (Johnson *et al.*, 2013). Although the safety profile and clinical use of rapamycin is well documented, the associated immunosuppressive effects, reduced wound healing and hyperlipidaemia may limit its use for mitochondrial disease (Johnson *et al.*, 2013). In keeping with this, recent evidence in patient cell lines also showed benefit from the partial inhibition of intra-mitochondrial translation using cycloheximide, and the inhibition of autophagy by lithium chloride (Peng *et al.*, 2015).

### Inhibiting opening of the mitochondrial permeability transition pore

Opening of the mitochondrial permeability transition pore (PTP) is thought to deplete pyridine nucleotides thus impairing mitochondrial oxidative respiration. Cyclosporin A inhibits the mitochondrial PTP and has been shown to induce muscle regeneration, improve mitochondrial function, and suppress muscle apoptosis (Merlini *et al.*, 2008) in both a murine model of collagen VI-deficiency myopathy and five patients with the same disorder. These findings lend support to the use of cyclosporin A in other mitochondrial disorders such as LHON, where a clinical trial is currently recruiting patients (Milea, 2014). However, like rapamycin, this widely used immunosuppressant may have a non-acceptable side effect profile in chronic use.

## Stem cell approaches

**Figure 5:**
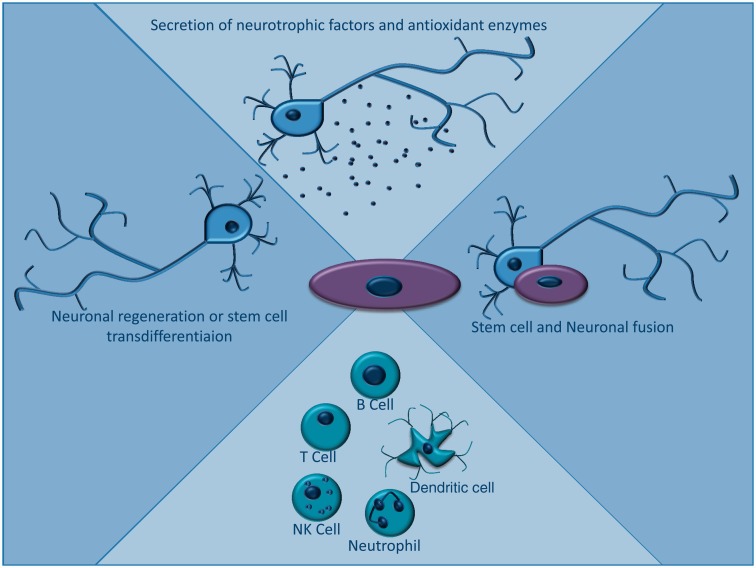
**Mechanism of action of stem cell therapies.** Various mechanisms have been described for the therapeutic action of stem cells for neurodegenerative conditions. These include secretion of neurotrophic factors and antioxidant enzymes such as superoxide dismutase, modulation of the immune system, regeneration of neurons and more controversially, stem cell transdifferentiation into neurons. Recently mesenchymal stem cells have been demonstrated to fully fuse with native cells to form heterokaryons or partially fuse via junction formation and transfer cellular organelles and factors.

Substantial preclinical, and increasing clinical evidence exists to support the use of stem cell therapies in several neurological disorders where mitochondrial dysfunction plays a role (e.g. Parkinson’s disease) (Lunn *et al.*, 2011). The proposed therapeutic mechanisms of stem cells are summarized in Fig. 5 (Parr *et al.*, 2007; Kemp *et al.*, 2010, 2014). Although not specific to mitochondrial dysfunction, harnessing these approaches could be of therapeutic benefit. Unfortunately the relative lack of animal models for primary mitochondrial disorders (Farrar *et al.*, 2013) has limited investigation of stem cell therapies for mtDNA disease.

### Exogenous stem cell therapy for nuclear DNA mutations

A number of case reports and small case series, discussed below, have described the effects of allogenic stem cell treatment in patients with MNGIE (Table 2) (Hirano *et al.*, 2006; Hill *et al.*, 2009; Lenoci *et al.*, 2011; Filosto *et al.*, 2012; Sicurelli *et al.*, 2012; Hussein, 2013). The rationale behind the treatment is based on work where haemo/peritoneal dialysis or platelet transfusions reduced circulating levels of toxic thymidine, presumably by introducing cells with normal thymidine phosphorylase activity (Moran *et al.*, 2008; Bax *et al.*, 2013; Hussein, 2013). Restoring thymidine phosphorylase activity in the bone marrow has been shown to reduce toxic thymidine levels post-transplantation, although the results were highly variable (Hirano *et al.*, 2006; Hill *et al.*, 2009; Halter *et al.*, 2011; Lenoci *et al.*, 2011; Filosto *et al.*, 2012; Sicurelli *et al.*, 2012; Hussein, 2013), and there is no convincing evidence that objective clinical benefit was actually achieved.

Interestingly, a recent retrospective analysis of all the 24 patients with MNGIE known to undergo a haematopoietic stem cell transplantation between 2005 and 2011 reported that in the nine survivors, thymidine phosphorylase activity rose from undetectable to normal levels (Halter *et al.*, 2015).

Two trials at Columbia University, USA are currently recruiting; one to map the natural history study of MNGIE (Hirano, 2015a) and the other to asses the safety of stem cell transplant for MNGIE in a proposed 12 patients (Hirano, 2015b). These should help inform future clinical trial design and begin to address safety concerns highlighted by a consensus report published by Halter *et al.* (2011).

### Endogenous stem cells for mitochondrial DNA mutations

There has been ongoing interest in the phenomenon of ‘gene shifting’ as a treatment for mitochondrial myopathy (Clark *et al.*, 1997; Spendiff *et al.*, 2013). In patients with heteroplasmic mtDNA mutations, the muscle satellite cells (myogenic stem cells) typically contain less mutated mtDNA than mature post-mitotic skeletal muscle fibres.

Pharmacological methods and resistance exercise to induce satellite cell proliferation and fusion with mature muscle to deliver wild-type mtDNA into mature muscle (Spendiff *et al.*, 2013) have been demonstrated to reduce mtDNA load in mature muscle (Clark *et al.*, 1997) and improve biochemistry. However, there is currently no evidence of clinical benefit (Andrews *et al.*, 1999). Recent work using somatic cell nuclear transfer adds weight to this approach, showing the correction of the metabolic disturbance with minimal disruption of nuclear-mitochondrial communication using induced pluripotent stem cells, albeit *in vitro* (Ma *et al.*, 2015).

A series of collaborative trials for patients with mtDNA mutations (comprehensively reviewed) (Kerr, 2013) demonstrated that endurance training increased work capacity and mitochondrial enzyme activity, but did not reduce heteroplasmy. Thus the benefits in this context were unlikely to be attributable to gene shifting and may simply reflect increased oxygen delivery through the development of capillary networks. Moreover, there may be a finite capacity for repair following induction of satellite cell proliferation by these methods; raising concerns about long-term sequelae. Finally, any potential benefits are most likely help the minority of patients with an isolated mitochondrial myopathy. Despite these concerns, there is currently an ongoing crossover trial investigating exercise versus inactivity in a cohort of patients with mitochondrial myopathy (Haller, 2012). This will hopefully provide a definitive answer to the role of exercise training in this context.

## Conclusion

The past 5 years have seen several new approaches developing through our understanding of the molecular pathogenesis of mitochondrial diseases. For mtDNA disorders, the early clinical studies attempting to harness gene-shifting some 15 years ago have not really progressed beyond early open labelled studies—probably because the likely clinical impact is limited using current approaches. Although intriguing, other molecular approaches directed against mtDNA disease are very much at the preclinical stage, and will require substantial development to improve efficacy and ensure there is no substantial risk of toxicity before human trials. From a clinical perspective, nuclear-genetic enzyme defects show the greatest promise. Stem cell therapy is already being used in specific contexts, and its efficacy and safety being evaluated, and gene therapy trials in mouse models show clear benefits. Unfortunately, each one of these rare genetic diseases may require their own proprietary approach, and the impact needs to be evaluated long-term. Small molecules are attractive because they have the potential to provide a more generic solution applicable across the mitochondrial disease spectrum, and a greater understanding of cell signalling pathways opens up several unexpected disease targets. For some of these drugs, clinical evaluation is imminent, particularly for those being repurposed or repositioned drugs such as bezafibrate. It is critical at this stage that laboratory and clinical scientists work closely with patient organizations to ensure that the ultimate aims of therapy will actually tackle issues that are important to patients. Given limited resources, this will ensure that new treatments improve quality of life—a prerequisite if these treatments are going to be adopted by healthcare systems worldwide.

## Funding

G.P. is the recipient of a Bisby Fellowship from the Canadian Institutes of Health Research D.B. is the recipient of a Kennedy Scholarship. P.F.C is a Wellcome Trust Senior Fellow in Clinical Science (101876/Z/13/Z), and a UK NIHR Senior Investigator, who receives support from the Medical Research Council Mitochondrial Biology Unit (MC_UP_1501/2), the Wellcome Trust Centre for Mitochondrial Research (096919Z/11/Z), the Medical Research Council (UK) Centre for Translational Muscle Disease (G0601943), EU FP7 TIRCON, and the National Institute for Health Research (NIHR) Biomedical Research Centre based at Cambridge University Hospitals NHS Foundation Trust and the University of Cambridge. The views expressed are those of the author(s) and not necessarily those of the NHS, the NIHR or the Department of Health.

## Literature search strategy

References for this Review were identified by searches of PubMed for English language articles published between 1970 and September 2015 and references of relevant articles and our personal libraries. The search terms ‘mitochondrial disease’, ‘mitochondrial disorder’, ‘mitochondrial encephalopathy’, ‘Leber’s hereditary optic neuropathy’, ‘mitochondrial neurogastrointestinal encephalopathy’, ‘mitochondrial encephalomyopathy, lactic acidosis and stroke-like episodes’, ‘neurogastrointestinal encephalopathy’, ‘myoclonic epilepsy with ragged red fibers’, ‘Kearns Sayre syndrome’, ‘neuropathy, ataxia, and retinitis pigmentosa’, and ‘inborn error metabolism’ were individually combined with ‘treatment’, ‘therapy’, ‘stem cell’, ‘stem cell transplant’, ‘bone marrow transplant’, ‘genome’, ‘genetics’ and ‘gene therapy’ were used. The final reference list was generated based on relevance to the topics covered in this Review.

## Abbreviations

AAV =: adeno-associated viral vector
Cas9 =: clustered regularly interspaced short palindromic repeats associated protein 9
COX =: cytochrome *c* oxidase
LHON =: Leber hereditary optic neuropathy
MNGIE =: mitochondrial neurogastrointestinal encephalopathy
PARP =: poly ADP (adenosine diphosphate-ribose) polymerase
PGC-1α =: peroxisome proliferator-activated receptor gamma coactivator-1-alpha
PPARs =: peroxisome proliferator-activated receptors
TALENs =: transcription activator-like effectors nucleases
ZFN =: zinc finger nuclease
